# Input and Processing Factors Affecting Infants’ Vocabulary Size at 19 and 25 Months

**DOI:** 10.3389/fpsyg.2018.02398

**Published:** 2018-11-30

**Authors:** Jae Yung Song, Katherine Demuth, James Morgan

**Affiliations:** ^1^Department of Linguistics, University of Wisconsin-Milwaukee, Milwaukee, WI, United States; ^2^Department of Linguistics, Centre for Language Sciences, ARC Centre of Excellence in Cognition and its Disorders, Macquarie University, Sydney, NSW, Australia; ^3^Department of Cognitive, Linguistic and Psychological Sciences, Brown University, Providence, RI, United States

**Keywords:** language acquisition, individual differences, vocabulary size, infant-directed speech, word recognition

## Abstract

This study examined the relative contributions of three factors to individual differences in vocabulary development: the acoustic quality of mothers’ speech, the quantity of mothers’ speech, and infants’ ability to recognize words. To examine the quality and quantity of mothers’ speech, recordings were collected from 48 mothers when their infants were 17 months old. Infants’ ability to recognize words was gauged by their performance in a perception experiment at 19 months. We examined the relationship between these measures and infants’ vocabulary size at 19 and 25 months. The quantity of mothers’ speech accounted for the greatest amount of variance in infants’ vocabulary size at 19 months; infants’ ability to recognize words followed next. At 25 months, when mothers’ speech alone is presumably no longer the primary input for infants, infants’ ability to recognize words at 19 months was a better predictor of vocabulary size. The acoustic quality of mothers’ speech was not correlated with infants’ vocabulary size at either age. The findings highlight the importance of considering multiple factors that contribute to early word learning, providing a better understanding of the mechanisms underlying the facilitation process.

## Introduction

It is easy to find substantial individual differences among children in early language development. Just as children produce their first words at different ages, they display individual variation in the rate of many different aspects of language development such as phonology (Leonard et al., 1980; Vihman and Greenlee, 1987), syntax (Brown, 1973) and vocabulary (Bates et al., 1991; Hart and Risley, 1995). For example, in terms of productive vocabulary size assessed by parental report on the MacArthur-Bates Communicative Development Inventory (MCDI) (Dale and Fenson, 1996), 16-month-olds in the lowest 10th percentile produce no words, whereas infants of the same age in the top 10th percentile produce at least 154 words (Bates et al., 1995). At 24 months, the gap is equally dramatic, ranging between 89 and 534 words. One of the major challenges in the study of language acquisition is therefore to identify the factors that contribute to individual variation in language development. The present study was motivated by the question: What factors account for individual differences in early vocabulary size? Hart and Risley (1995) suggested that the vocabulary size and growth that were observed for 3-year-olds still predicted receptive and productive skills at age 9–10, thereby potentially influencing academic performance at school. Their results highlight the importance of understanding the factors that contribute to individual variation in early vocabulary size.

Several studies have shown positive correlations between various measures of the quantity of infant-directed (ID) speech and infants’ vocabulary size. For example, Hart and Risley (1995) showed that the average child in an American professional family heard significantly more word tokens and types than the average child in a working-class family, who heard significantly more than the average child on welfare. What was striking was that the children’s vocabulary size at 3 years of age closely mirrored these differences in parental speech input. From these findings, Hart and Risley (1995) suggested that it was parents’ quantity of speech, rather than their social class or income *per se*, that directly predicted their children’s vocabulary development. Similarly, Hoff and Naigles (2002) showed positive correlations between the number of word types and tokens and mean length of utterance (MLU) in mothers’ speech when their children were 18–29 months, and their children’s vocabulary size measured 10 weeks later. Huttenlocher et al. (1991) also demonstrated that mothers who used a greater number of word tokens had children with faster vocabulary growth between 14 and 26 months. Pan et al. (2005) showed that mothers’ number of word types predicted the growth of children’s word types between 14 and 36 months of age, whereas the number of word tokens did not. Their findings suggested that the number of word types was a better predictor of vocabulary growth than word tokens, at least for children from low-income families. Taken together, these studies showed that the quantity of mothers’ speech is a powerful predictor of early vocabulary development.

Another factor that has received much attention is individuals’ speech processing abilities (for reviews on this topic, see: Kuhl et al., 2005; Saffran and Graf Estes, 2006; Cristia et al., 2014). For instance, 25-month-olds who were faster and more accurate in a word recognition task were also more advanced in vocabulary and grammatical development from 12 to 25 months (Fernald et al., 2006). Similarly, 7- to 12-month-olds’ ability to segment words in the speech stream was positively correlated with their expressive vocabulary size at 24 months (Newman et al., 2006). Furthermore, the performance of 6-month-olds in a phonetic discrimination task was linked with their vocabulary size at 13, 16, and 24 months (Tsao et al., 2004). Bernhardt et al. (2007) found that infants who were more successful in a word-object association task at 17–20 months were also more advanced in both word comprehension and production after 1–2 years. A similar association between children’ productive vocabulary size and their processing efficiency was also found in an event-related potential (ERP) study (Torkildsen et al., 2008). These results suggest that infants who demonstrate more efficient skills in processing speech in experimental settings also use these abilities to learn words in the real world. This may account in part for their reported faster vocabulary development.

So far, the role of the quantity of ID speech and infants’ ability to process speech has been almost always studied separately. However, more recently, Newman et al. (2016) demonstrated that variation in infants’ vocabulary size was best explained when the two factors were considered simultaneously. When considered together, parents’ repetition of words and infants’ segmentation ability at 7 months of age explained nearly 11% of the variation in 2-year-olds’ vocabulary size. This study suggested that it is important to consider multiple factors simultaneously.

In addition to the quantity of ID speech and infants’ ability to process speech, researchers have also raised the importance of considering the acoustic quality of ID speech in investigating vocabulary development (Huttenlocher et al., 1991; Newman et al., 2006). In contrast to adult-directed (AD) speech, ID speech is characterized by slower speaking rate, higher pitch, exaggerated pitch contours, and careful articulation of speech sounds (Fernald et al., 1989; Kuhl et al., 1997; Davis and Lindblom, 2001). It has been long suggested that the exaggerated acoustic properties of ID speech may facilitate language acquisition. For example, Thiessen et al. (2005) showed that infants were able to use statistical information to distinguish words from partial words after exposure to ID speech, but not after hearing AD speech. Ma et al. (2011) showed that 21-month-olds reliably learned novel words presented within sentences only when they heard them in ID speech. Unlike 21-month-olds, 27-month-olds were able to learn new words after hearing AD speech.

If there are certain acoustic properties of ID speech that facilitate aspects of the word learning process, we may consider that mothers who use these properties provide good quality acoustic input to infants. Thus, one logical question to ask would be whether the acoustic quality of early language experience accounts for some of the individual variation found in language development. Thus far, little evidence is available concerning the relationship between the acoustic quality of mothers’ speech and their infants’ language skills. Liu et al. (2003) showed that there was a large variability in the degree of vowel hyper-articulation among Chinese mothers, and that the mothers who hyper-articulated vowels had 6–8- and 10–12-month-olds who performed better in discriminating the affricate-fricative contrast in Chinese. Similarly, Cristià (2011) showed that English-learning 5 and 13-month-olds whose caregivers produced more extreme /s/ were better able to discriminate /s/ from /∫ /. Note that both of these studies examined infants’ speech discrimination skills. We are aware of only one study, published recently, that investigated the relationship between the acoustic quality of ID speech and infants’ expressive language outcomes. Hartman et al. (2017) examined whether measures of mothers’ vowel clarity at 10–11, 18, and 24 months were related to 24-month-olds’ expressive and receptive language outcomes. Children were divided into two groups depending on the size of mothers’ vowel space area. The results showed that children who had mothers with larger vowel space at 18 months had significantly better expressive and receptive language outcomes.

Our review of the literature suggests that there are important gaps in the literature regarding our understanding of the factors contributing to early vocabulary development. First, very few studies have addressed or controlled the acoustic quality of mothers’ speech. Furthermore, because each of the previous studies has focused on only a single factor, there is limited understanding of the relative contributions of the acoustic quality of mothers’ speech, the quantity of mothers’ speech, and infants’ ability to process speech.

The goal of the current study was therefore to provide a better understanding of the relative contributions of these three factors to infants’ vocabulary size. In order to examine the quality and quantity of mothers’ speech, mothers were recorded speaking to their infants when they were 17 months old. Two months later, when the infants were 19 months old, the same mothers and their infants returned to the lab and the infants participated in a word recognition test. Infants’ performance in this test served as an indicator of their ability to recognize words. Infants’ vocabulary size was assessed by parental report on the MCDI when they were 19 and 25 months. We were particularly interested in examining infants’ vocabulary development around the ‘vocabulary spurt’ (Goldfield and Reznick, 1990) that typically begins at approximately 18 months.

Based on the results from previous studies, we predicted there would be a significant correlation between the quantity of mothers’ speech and infants’ vocabulary size, as well as between infants’ ability to recognize words and their vocabulary size. The scarcity of relevant literature makes it difficult to formulate firm predictions regarding the effect of the acoustic quality of mothers’ speech. However, studies have shown that infants’ speech discrimination skills are related to their expressive vocabulary size, and there is emerging evidence suggesting that the quality of ID speech (as well as the quantity of ID speech, as will be discussed in the “Discussion” section) is related to infants’ speech discrimination skills. Considering the links, it would seem reasonable to assume that the quality of ID speech may be related to infants’ expressive vocabulary size as well. In particular, slow speaking rate and expanded vowel space of ID speech have been shown to enhance 19-month-olds’ ability to recognize words (Song et al., 2010). If the experience of successful word recognition accumulates over time, this would help infants build their phonological representations of words more efficiently as compared to infants who fail to recognize the word at times. This hypothesis is consistent with the view that lexical representations are built up gradually and become more robust through experience (Fernald et al., 2006). If so, mothers who use slow speaking rate and vowel hyper-articulation would have infants who are more successful in acquiring words than mothers who provide relatively poor quality of acoustic input to infants. We would predict, then, that the acoustic quality of mothers’ speech would contribute to individual differences in vocabulary size.

## Materials and Methods

### Participants

Forty-eight mothers and their 17-month-old English-monolingual infants (21 females, 27 males; mean age: 16.9 months, range: 16.1–18 months), recruited in Providence, Rhode Island, participated in recordings of ID speech. Approximately 2 months later, the same mothers and their 19-month-old infants (mean age: 19.4 months, range: 18.6–20.7 months) returned to the lab and the infant participated in a word recognition experiment. An additional 22 mother-infant dyads participated in recordings but were excluded for various reasons: 14 due to the failure to schedule the second visit for the word recognition test, 5 due to fussiness during the word recognition test, 3 due to experimental error. After completing the recording, the mother was asked to fill out a short questionnaire. The survey revealed that the majority of the mothers were the primary caretakers of their children. Only 5 out of the 48 mothers had jobs (4 full-time jobs and 1 part-time job). The stay-at-home mothers responded that, on average, they spent around 10 hours a day with their children. Most of the working mothers replied that they interacted approximately 5 hours a day with their children. In addition, the majority of the participants (47 out of 48, or 98%) were white. This study was reviewed and approved by the Institutional Review Board of Brown University. All mothers provided written informed consent prior to participation of their infants, as well as written informed consent for their own participation.

### MacArthur-Bates Communicative Development Inventories (MCDI)

To measure infants’ vocabulary size, mothers were asked to check the words that their infants produced, from a list of 680 words in MCDI (toddler form, full version). The questionnaires were sent out twice, when the infants were 19 and 25 months old. At 19 months, 42 out of 48 mothers returned their questionnaires, and at 25 months, 27 mothers out of 48 mothers returned their questionnaires. There were 25 mothers who returned their MCDIs at both 19 and 25 months.

### Recording and Transcription Procedures

To examine the acoustic quality and quantity of mothers’ speech, spontaneous speech samples were taken from the mothers when their infants were 17 months old. Recordings were made in a quiet testing room using an Olympus DM-20 digital voice recorder. During the recording, the mother wore a lavalier microphone pinned to her collar and played with her infant in a natural environment for 15 min. Toys were provided to elicit the mother’s production of the target words *box*, *sheep,* and *shoes*. These words were chosen because they were familiar to the children, and because we wanted to calculate the vowel space using the point vowels /a/, /i/, and /u/.

After the recording session, the audio recording was downloaded onto a computer and digitized at a sampling frequency of 44.1 KHz with 16-bit resolution. Then, using the CHAT transcription system (MacWhinney, 2000), four trained coders orthographically transcribed the mother’s utterances. Employing the CHAT transcription format enabled us to automatically calculate the number of word types and tokens, as well as MLU. Also, each sentence in the transcription was linked to a corresponding sound file which was then directly sent to acoustic analysis software Praat (Boersma and Weenink, 2014).

#### Analysis of the Quantity of Mothers’ Speech

Four measures were used to gauge the quantity of mothers’ speech during the 15-min recording sessions: the number of tokens, the number of types, token/type ratio, and MLU. First, in order to examine how talkative mothers were, we calculated the total number of words (tokens) produced by each mother. Second, to evaluate the lexical diversity in mothers’ speech, we examined the total number of different words (types) produced by each mother. Third, to examine the relationship between the number of tokens and the number of types, we looked at the token/type ratio, which indicates how many times each type is repeated. Fourth, to examine the length or syntactic complexity of mothers’ utterances, MLU was calculated in words for each mother.

We made several decisions regarding what counted as a token and what counted as a type. Contractions (*there’s*), concatenatives (*wanna*), and compound words (*shoebox*) were all counted as one token. Exclamations (*wow*) and onomatopoetic words (*baa*) were also included in the count of types as they were considered as an important aspect of mother–child interactions. Morphologically inflected variants of a word were counted as different word types (*like, likes, liked*: three word types), as were alternative forms of a word (many of which were diminutives) (*sheep, sheepy*: two word types).

Type, token, token/type ratio, and MLU calculations were carried out for the utterances that were sampled based on the presence of a target word. In total, 3393 utterances from 48 mothers contained at least one of the three target words and were included in the analysis. Because the participants were asked to play using the three toys provided, their activities during a recording session were centered on the three toys, and mothers’ utterances reflected that. Therefore, it was assumed that the utterances containing the three target words were representative of the mothers’ overall utterances during recording sessions. To verify this assumption, recordings of 12 out of 48 mothers were randomly selected and all 3846 utterances from the 12 mothers were transcribed. We then examined the correlation between the measures made on the basis of the utterances containing the target words and those made on all utterances. For all four measures, the results showed high correlation between the two: the number of word types, *r(10)* = 0.74, *p* < 0.01, the number of word tokens, *r(10)* = 0.70, *p* = 0.01, token/type ratio, *r(10)* = 0.85, *p* < 0.01, and MLU, *r(10)* = 0.72, *p* < 0.01. This suggested that the utterances containing the target words were representative of how mothers generally spoke during a 15-min recording session. Thus, we report here the numbers based on the sampled utterances.

#### Analysis of the Acoustic Quality of Mothers’ Speech

There were four measures of maternal acoustic quality. First, average speaking rate (the number of syllables per second) was calculated for each of the mothers. Second, mean vowel space was calculated in order to examine the degree of vowel clarity in each mother. The vowel space area was defined as the area of the triangle formed by the average first (F1) and second (F2) formant frequencies (measured at the vowel midpoint) of /a/, /i/, and /u/ in the target words *box*, *sheep*, and *shoes*. The vowel space area was calculated using a general mathematical formula for calculating the area of a triangle when one knows the coordinates of the three vertices of a triangle. The specific formula is as follows: Vowel space area = |{F1i × (F2a – F2u) + F1a × (F2u y F2i) + F1u × (F2i – F2a)}/2| (adapted from Liu et al., 2003). Here, F1i represents the F1 of vowel /i/, F2a represents the F2 of vowel /a/, and so forth. Lastly, we calculated average fundamental frequency (F0) and average F0 range over utterances for each mother. To compute F0 range, the maximum and minimum F0 values of each utterance were obtained and the difference was calculated. F0 values were first automatically extracted using WaveSurfer (Sjölander and Beskow, 2000) and then manually inspected. Praat (Boersma and Weenink, 2014) was used for the temporal measure (i.e., speaking rate) and WaveSurfer was used to measure F1, F2, and F0 values.

Because we wanted to control for the effect of utterance-level prosody on word production, we divided all utterances into two categories depending on where the target word occurred within the utterance: utterance-medial vs. utterance-final. In the present study, all utterance-medial words were non-clause final. Clause-final words (e.g., *You like these shoes, don’t you?*) were excluded from the analyses (220 utterances). Utterance-final words and the initial word of the following utterances were separated by pauses of at least 100 ms, with the majority of the pauses over 300 ms. We excluded utterances that were immediately followed by other utterance (73 utterances). These excluded utterance-final words were often accompanied by rising intonation and typically formed one breath group with the following utterances (e.g., *Let’s open the blue box. What’s in there?*).

We then examined a minimum of 5 to a maximum of 10 utterances with good acoustic quality in each position for each target word during a session. This decision was made on the basis that the number of word repetitions elicited from each speaker tends to not exceed 10 in speech production studies involving acoustic analysis, including many studies on ID speech (e.g., Kuhl et al., 1997; Uther et al., 2007). As a result, between 30 and 60 utterances (5–10 utterances × 2 positions × 3 target word) were analyzed for each mother in the present study. The final data set used in the acoustic analysis included 1614 utterances.

### Word Recognition Test

The infants participated in a word recognition test at 19 months of age. Using the intermodal preferential looking procedure, infants were presented with pictures of the target (e.g., *cup*) and a distractor (e.g., *hat*), and received audio stimuli asking them to find the target (*Where is the cup?*). The stimuli were pre-recorded in typical ID speech style by a female speaker. As shown in Table 1, there were 12 target-distractor pairs. The target and a distractor objects were chosen to be easily picturable and highly familiar to 19-month-olds, as indicated by the proportion of infants reported understanding the words in the MCDI. Infants’ eye movement behavior during the test trial was video-recorded and then coded offline using Supercoder software (Hollich, 2003).

**Table 1:** Word pairs used in the word recognition test.

	Target	Proportion	Distractor	Proportion
(1)	Bird	79	Dog	88
(2)	Block	69	Sock	85
(3)	Bunny	61	Puppy	61
(4)	Car	93	Ball	93
(5)	Cat	76	Hat	61
(6)	Cup	86	Book	90
(7)	Door	79	Chair	64
(8)	Flower	68	Apple	74
(9)	Hand	64	Bed	68
(10)	Kitty	78	Cookie	83
(11)	Spoon	75	Balloon	82
(12)	Truck	67	Duck	79

*The proportion indicates the percentage of 16-month-old infants reported to understand the words in the MCDI.*

Before infants received each test trial, they received a salience trial where they were presented with pictures of the target and a distractor while listening to a ‘neutral’ sentence such as “What are these?”. The salience trial served to create the expectation that something would appear on each screen and introduced the video events before the infant had to find the match for the audio stimulus in the test trial, as well as to provide a baseline measure of the relative visual salience of each of the objects. During the salience trial, the proportions of infants’ looking time to the target were 54% (*SD* = 11.05). The proportions of looking time to the target object significantly increased to 70% (*SD* = 11.74) during the test trial. A one-sample *t*-test indicated that the amount of increase in the proportion looking time to the target from the salience trial to the test trial was significantly different from chance, *t*(47) = 6.60, *p* < 0.001, suggesting that children recognized the target word during the test trial.

To examine how accurately and quickly individual infants recognized the target words during the test trial, two measures were made within 2.5 s after the target word was played (e.g., *Where is the cup?*): the proportion of looking time to the target and the latency of the first look to the target. The proportion of looking time to the target was calculated by dividing the looking time to the target object by the sum of the looking time to the target and a distractor. Response latency was defined as the time taken for infants to first look at the target within 2.5 s after the target word offset, regardless of whether they looked directly at the target picture after they listened to the test stimuli, or whether they incorrectly looked at the distractor first and then shifted to the target.

### Reliability Check

All data were coded by the first author, and a randomly chosen 10% of the total data (5 out of 48 mothen–child dyads) were recoded by trained research assistants to evaluate inter-coder reliability. One measure (vowel space) was recoded by the first author approximately 2 years after the original measurement and intra-coder reliability was assessed. Pearson *r* correlation coefficients between the measurements of the original and recoded data were over 0.95 for all measures except for one measure, latency of the first look to target, for which the correlation coefficient was 0.80. All correlations were significant at *p* < 0.001, suggesting high inter- and intra- coder reliability.

## Results

The dependent variable in the present study was infants’ productive vocabulary size measured at 19 and 25 months by parental report on the MCDI. The independent variables included the four measures of the acoustic quality of mothers’ speech (speaking rate, vowel space, F0, F0 range), four measures of the quantity of mothers’ speech (number of tokens, number of types, token/type ratio, MLU), and two measures of infants’ ability to recognize words (proportion of looking time to the target, latency of the first look to the target). In the following sections, we first present descriptive statistics of the dependent and independent variables. Then we examine the associations between infants’ vocabulary size and the various measures of the acoustic quality of mothers’ speech, the quantity of mothers’ speech, and infants’ ability to recognize words.

### Infants’ Vocabulary Size

On average, mothers replied that their 19-month-olds produced 105 words (range: 4–545, *SD* = 114) and 25-month-olds produced 304 words (range: 23–680, *SD* = 187). At 25 months, one child was at ceiling, producing all 680 words in the MCDI. There was a strong correlation between infants’ vocabulary size at 19 and 25 months, *r(23)* = 0.75, *p* < 0.001.

### The Acoustic Quality of Mothers’ Speech

On average mothers produced 4.43 syllables/second, with a range of 2.92–6.16 syllables/second (*SD* = 0.68). As shown from the wide range and a high standard deviation, a considerable amount of variability was found among mothers; notice that the fastest speaking mother spoke over twice as fast as the slowest speaking mother. The average vowel space area in mothers’ speech to 17-month-olds was 271,855 Hz^2^, with a large amount of variability in the vowel space among the individual mothers (range: 92,294–657,604 Hz^2^, *SD* = 132,116). Average F0 in mothers’ speech to 17-month-olds was 263 Hz (range: 195–360 Hz, *SD* = 28), and average F0 range was 247 Hz (range: 147–372 Hz, *SD* = 46).

### The Quantity of Mothers’ Speech

The average number of tokens mothers produced was 396 (range: 90–735, *SD* = 182). The average number of different word types was 105, with a range of 31–182 (*SD* = 34). An examination of the token/type ratio revealed that on average, mothers repeated each word type 3.7 times, with a range of 2.19–5.82 (*SD* = 0.88). The average MLU in words was 5.56 (range: 3.59–7.81, *SD* = 0.98), meaning that mothers’ utterances to their 17-month-olds were on average 5.56 words long. We also examined MLU in morphemes using the MOR and POST procedures in CLAN (MacWhinney, 2000). Consistent with previous findings (Parker and Brorson, 2005), MLU in words and MLU in morphemes (mean: 6.32, range: 4.37–8.88 *SD* = 1.03) were highly correlated in the present study, *r*(45) = 0.95, *p* < 0.001. In our analysis, we used MLU in words.

### Infants’ Ability to Recognize Words

The average proportion of time that 19-month-olds spent looking at the target during the test trial was 70% (range: 48–97, *SD* = 11.74). On average, it took 0.69 s for infants to first look at the target (range: 0.26–1.63 s, *SD* = 0.29).

In sum, there was a considerable amount of variability in both the acoustic quality and the quantity of mothers’ speech to their 17-month-olds, as well as in infants’ ability to recognize words at 19 months. The existence of large variability raises the possibility that some of these factors may correlate with individual differences in vocabulary size. We examine this issue below.

### Factors Predicting Infants’ Vocabulary Size at 19 Months

We begin by examining simple correlations between each independent variable and infants’ vocabulary size. As shown in Table 2, there were four variables that were individually correlated with infants’ vocabulary size at 19 months: number of word tokens, number of word types, proportion of looking time to the target, and latency to target. None of the measures of acoustic quality of mothers’ speech was correlated with infants’ vocabulary size. Token/type ratio was also not significant, suggesting that simply repeating words was less important than producing a variety of words. Unlike previous studies showing a correlation between mothers’ MLU and children’s vocabulary size (Hoff and Naigles, 2002), no significant correlation was found between the two in the current study. These results from simple correlation analyses provided a baseline for the relationship between each variable and infants’ vocabulary size, showing that there were multiple factors that accounted for infants’ vocabulary size.

**Table 2:** Results of simple correlation analyses between vocabulary size at 19 months and different independent variables.

	Independent variables	*r*-value	*p*-value
Acoustic quality	Speaking rate	*r*(39) = 0.09	*p* = 0.57
of mothers’	Vowel space	*r*(26) = −0.02	*p* = 0.91
speech	F0	*r*(39) = −0.17	*p* = 0.30
	F0 range	*r*(39) = −0.11	*p* = 0.48

Quantity of	Number of tokens	*r*(38) = 0.31	*p* < 0.05
mothers’	Number of types	*r*(39) = 0.40	*p* < 0.01
speech	Token/type ratio	*r*(38) = 0.06	*p* = 0.72
	MLU	*r*(39) = 0.19	*p* = 0.22

Infants’ ability	Proportion looking to target	*r*(40) = 0.45	*p* < 0.01
to recognize words	Latency to target	*r*(40) = −0.35	*p* < 0.05

Next we conducted a hierarchical multiple regression analysis to examine the relative contributions of each of the quality of mothers’ speech, the quantity of mothers’ speech, and infants’ ability to recognize words in predicting infants’ vocabulary size at 19 months. In the hierarchical multiple regression analysis, the variables are entered into the regression in a series of blocks or groups. Thus, the hierarchical approach is appropriate when the researcher has *a priori* ideas about how the variables go together to predict the dependent variable (Leech et al., 2008). In order to determine the order of the three factors (quality of mothers’ speech, quantity of mothers’ speech, and infants’ ability to recognize words – all represented by multiple measures) to be entered into the regression, we first ran three separate multiple regression analyses on each of the factors. Then the factor whose regression model had a greater adjusted *R*^2^ was entered into a hierarchical multiple regression analysis first.

Results from the three separate multiple regression analyses showed that the combination of the variables of the quantity of mothers’ speech accounted for the greatest amount of the variance in infants’ vocabulary size, as indicated by the adjusted *R*^2^ value of 0.21, *F*(4,35) = 3.65, *p* < 0.05. The model of the infants’ ability to recognize words was also significant, *F*(2,39) = 5.19, *p* = 0.01, adjusted *R*^2^ = 0.17. In contrast, the model of the acoustic quality of mothers’ speech was not significant, *F*(4,23) = 1.04, *p* = 0.41, adjusted *R*^2^ = 0.01. Thus, the measures of the acoustic quality of mothers’ speech were not considered further in the hierarchical multiple regression analysis.

Two blocks of independent variables were entered into the hierarchical multiple regression: the quantity of mothers’ speech and infants’ ability to recognize words. The four measures of the quantity of mothers’ speech were entered first as a block because they had a greater adjusted *R*^2^. Each of the four variables were entered stepwise, as we wanted to know which of the four variables were most significant predictors of infants’ vocabulary size. The second block added the two measures of infants’ word recognition ability. Again, the variables within this block were entered stepwise.

The results from the hierarchical multiple regression analysis showed that the variables of the quantity of mothers’ speech significantly predicted infants’ vocabulary size at 19 months, *F*(1,38) = 9.75, *p* < 0.01, *R*^2^ = 0.20. The only variable that significantly contributed to the model was the number of word types in mothers’ speech (see Table 3). When the variables of infants’ ability to recognize words were added, they significantly improved the prediction, *F*(1,37) = 6.55, *p* < 0.05, *R*^2^ change = 0.12. Thus, the effect of infants’ ability to recognize words was significant even when controlling for the effect of the quantity of mothers’ speech, but as indicated by *R*^2^, the quantity of mothers’ speech accounted for the greater proportion of variance in infants’ vocabulary size than infants’ ability to recognize words. As shown in Table 3, the only variable of infants’ ability to recognize words that significantly contributed to the model was the proportion of looking time to the target. Both blocks of variables significantly predicted infants’ vocabulary size at 19 months, *F*(2,37) = 8.86, *p* < 0.01, adjusted *R*^2^ = 0.29.

**Table 3:** Hierarchical multiple regression analysis summary for the factors predicting infants’ vocabulary size at 19 months.

Variable	*R*^2^	*R*^2^ change	Coefficient B	*SE B*	β	*p*-value	95% CI for B	
							Lower	Upper
Step 1	0.20	0.20						
Number of types			1.59	0.51	0.45	<0.01	0.56	2.62
Constant			−59.82	56.72				

Step 2	0.32	0.12						
Number of types			1.35	0.48	0.39	<0.01	0.37	2.33
Proportion looking to target			3.41	1.33	0.35	<0.05	0.71	6.11
Constant			−277.01	100.06				

In sum, the hierarchical multiple regression analysis showed that the lexical diversity of mothers’ speech to 17-month-olds, and 19-month-olds’ proportion looking to the target in the word recognition task, were both significant predictors of infants’ vocabulary size at 19 months. On the other hand, none of the measures of acoustic quality of mothers’ speech was correlated with infants’ vocabulary size at 19 months. In the next section, we examine which of the variables predict infants’ vocabulary size at 25 months.

### Factors Predicting Infants’ Vocabulary Size at 25 Months

At 25 months, we examined data from the 27 mother–child dyads who returned the MCDI questionnaires. To investigate the predictors for infants’ vocabulary size at 25 months, again, we started from examining simple correlations between various individual variables of the three factors and infants’ vocabulary size. Only one of the variables was significant at 25 months: infants’ proportion looking to the target in the word recognition task (see Table 4 below). The effect of the number of types in mothers’ speech, which was significant at 19 months, was no longer significant.

**Table 4:** Results of simple correlation analyses between vocabulary size at 25 months and different independent variables.

	Independent variables	*r*-value	*p*-value
Acoustic quality	Speaking rate	*r*(24) = 0.21	*p* = 0.29
of mothers’	Vowel space	*r*(15) = −0.09	*p* = 0.73
speech	F0	*r*(24) = −0.12	*p* = 0.56
	F0 range	*r*(24) = −0.10	*p* = 0.62

Quantity of	Number of tokens	*r*(24) = 0.13	*p* = 0.54
mothers’	Number of types	*r*(24) = 0.19	*p* = 0.35
speech	Token/type ratio	*r*(24) = 0.02	*p* = 0.92
	MLU	*r*(24) = 0.07	*p* = 0.75

Infants’ ability	Proportion looking to target	*r*(25) = 0.44	*p* < 0.05
to recognize words	Latency to target	*r*(25) = −0.05	*p* = 0.82

The three separate multiple regressions were then computed on the three factors under examination: the quality and the quantity of mothers’ speech, and infants’ ability to recognize words. The combination of the variables of infants’ ability to recognize words significantly accounted for the variance in infants’ vocabulary size at 25 months, *F*(2,24) = 3.56, *p* < 0.05, adjusted *R*^2^ = 0.17. On the other hand, the models of the acoustic quality of mothers’ speech [*F*(4,12) = 0.22, *p* = 0.92, adjusted *R*^2^ = −0.24] and the quantity of mothers’ speech [*F*(4,21) = 0.64, *p* = 0.64, adjusted *R*^2^ = −0.06] were not significant. Thus, the measures of the acoustic quality and the quantity of mothers’ speech were not considered further in the hierarchical multiple regression analysis.

When the two variables of infants’ ability to recognize words were entered into the hierarchical multiple regression in a stepwise fashion, only the proportion of looking time to the target significantly contributed to the model (see Table 5). Jointly, the two variables significantly predicted infants’ vocabulary size at 25 months, *F*(1,25) = 5.89, *p* = 0.02, and accounted for 15.8% of the variance in vocabulary size, as indicated by the adjusted *R*^2^ value.

**Table 5:** Hierarchical multiple regression analysis summary for the factors predicting infants’ vocabulary size at 25 months.

Variable	*R*^2^	*R*^2^ change	Coefficient *B*	*SE B*	β	*p*-value	95% CI for B	
							Lower	Upper
Step 1	0.19	0.19						
Proportion looking to target			6.12	2.52	0.44	< 0.05	0.93	11.32
Constant			−132.11	182.59				

Thus, at 25 months, infants’ vocabulary size was significantly correlated with their proportion looking time to target in the word recognition task. However, neither the acoustic quality nor the quantity of mothers’ speech predicted infants’ vocabulary size at 25 months. Thus, by 25 months, infants’ own language abilities were a stronger predictor of vocabulary size than the quantity of their mothers’ speech.

As we have a smaller sample of infants’ vocabulary size information at 25 months, one might wonder if the absence of the effect of the quantity of mothers’ speech is simply due to the difference in sample population or size between 19 and 25 months. We think this is unlikely. For example, the average number of word types of the 42 mothers who returned the MCDI at 19 months was 108 (*SD* = 33.8). That of the 27 mothers who returned the MCDI at 25 months was the same (*M* = 108, *SD* = 34.5), suggesting that the samples are comparable. Furthermore, we conducted analyses of a subset of mother–child dyads at 19 months in order to compare results at 19 and 25 months with the same sample size (i.e., 27 dyads). Out of the 27 mothers examined at 19 months, 25 were the ones who returned the MCDI both at 19 and 25 months; 2 were randomly selected from the rest of mothers who sent the results only at 19 months. Results of simple correlation analyses of 27 mother–child pairs showed that 3 variables were significantly correlated with infants’ vocabulary size at 19 months: number of types [*r*(24) = 0.50, *p* = 0.01], MLU [*r*(24) = 0.43, *p* < 0.05], proportion looking to target [*r*(25) = 0.43, *p* < 0.05]. There was a tendency toward significance for the number of tokens, *r*(24) = 0.35, *p* = 0.08. Thus, most of the measures of the quantity of mothers’ speech were significantly correlated with infants’ vocabulary size at 19 months with only the 27 dyads, although none of them were significant at 25 months. Further multiple regression analyses showed that the model of the quantity of mothers’ speech accounted for the greatest amount of the variance in infants’ vocabulary size at 19 months, *F*(4,21) = 2.40, *p* = 0.08, adjusted *R*^2^ = 0.18. The model of the infants’ ability to recognize words followed the next, *F*(2,24) = 2.96, *p* = 0.07, adjusted *R*^2^ = 0.13. The model of the acoustic quality of mothers’ speech was least predictive, *F*(4,12) = 0.46, *p* = 0.77, adjusted *R*^2^ = −0.16. Because there was a trend toward significance for the models of the quantity of mothers’ speech and the infants’ ability to recognize words, we were interested in testing the effect of these factors in a hierarchical multiple regression analysis. When the variables of the quantity of mothers’ speech and infants’ ability to recognize words were entered into a hierarchical multiple regression in that order, only the model of the quantity of mothers’ speech was significant with the number of word types significantly contributing to the model, *F*(1,24) = 7.90, *p* = 0.01, adjusted *R*^2^ = 0.22. Overall, these results suggest that the quantity of mothers’ speech is a strong predictor of infants’ vocabulary size at 19 months and that the change in the effect of the quantity of mothers’ speech over time cannot be simply explained by the difference in sample size at 19 and 25 months.

## Discussion

The goal of this study was to determine the relative contributions of the quantity and quality of mothers’ speech and infants’ word recognition abilities in predicting infants’ expressive vocabulary size. The results showed that the number of word types in mothers’ speech accounted for the greatest amount of variance in infants’ vocabulary size at 19 months, but that this was superseded by infants’ own word recognition abilities at 25 months.

Although researchers have long proposed that the acoustic quality of ID speech may facilitate infants’ vocabulary development, currently it is not clear what the underlying mechanism of the facilitation is. To aggravate the problem, there have been almost no systematic investigations of this factor. So far, the only other available empirical data looking at infants’ expressive vocabulary size was provided by Hartman et al. (2017), who demonstrated a relationship between mothers’ vowel space at 18 months and infants’ vocabulary size at 24 months. The effect at 18 months was short-lived, and no relationship was found for mothers’ vowel space produced at earlier (10–11 months) and later (24 months) points. In the present study, we found no relationship between mothers’ vowel space produced at 17 months and infants’ vocabulary size at 19 and 25 months. There are several methodological differences between the two studies that make it challenging to produce direct comparisons. For example, unlike the present study, correlations were only visually inspected in Hartman et al. (2017). Although both studies had 15-min recording sessions, most of the acoustic measures employed in Hartman et al. (2017) (vowel duration, vowel variability) and the present study (speaking rate, average F0, F0 range) did not overlap. Nonetheless, both studies similarly showed that none of the acoustic measures were linked with infants’ vocabulary size, except for vowel space in Hartman et al. (2017). This lack of acoustic effect, together with the short-lived effect of vowel space in Hartman et al. (2017), suggest that even if the acoustic quality of ID speech play some role in the variation of infants’ vocabulary size, its role might be lesser compared to the other two factors.

If the acoustic quality of ID speech plays a lesser role in their infants’ vocabulary development, one possible explanation for this is that the adaptation to mothers’ voice occurs very early in life. For example, although 1-month-olds showed a strong listening preference for ID speech over AD speech when both were spoken by a woman unfamiliar to them, their general preference for ID speech disappeared when they listened to their own mothers’ voices (Cooper et al., 1997). Given that the familiarity with mothers’ voices occurs very early in life, there is the possibility that familiarity with the mothers’ voices might override the effects of mothers’ intelligibility on children’s word recognition process. Thus, when infants listen to an unfamiliar voice, as they did in past perception experiments (e.g., Song et al., 2010), their word recognition is highly affected by the acoustic properties of that voice, whereas the acoustic quality of their own mothers’ voices may be less influential.

However, although we found no significant correlation between the acoustic quality of mothers’ speech and their infants’ vocabulary size, we do not rule out the possibility that we have missed the critical window when the correlation is present. We also recognize that the acoustic quality of mothers’ speech could be positively correlated with other aspects of infants’ language development not explored in this study. Certainly, more research is warranted to confirm the link between the quality of ID speech and infants’ vocabulary size at different developmental ages/stages and to pinpoint the aspects of language development that are most influenced by the quality of ID speech.

The results of the present study showed that the number of word types in mothers’ speech at 17 months predicted infants’ vocabulary size at 19 months. However, this effect disappeared by 25 months. This is perhaps because the sources of speech input for infants increase with age as the scope of their interactions with others become broader. Thus, the quantity of *mothers*’ input alone may no longer be sufficient to predict infants’ vocabulary size by 25 months. It is informative to compare our findings with those of Pan et al. (2005), who examined predictors of growth in infants’ vocabulary between 14 and 36 months. Their results likewise showed that infants whose mothers used a greater number of word types showed faster vocabulary growth than infants whose mothers used a smaller number of word types. Interestingly, this effect was particularly strong around 24 months, though by 36 months, the effect had disappeared. There is a few months gap in infants’ ages between the present study and Pan et al. (2005) when the effect of maternal types is most pronounced. However, in both studies, the effect was particularly strong during the earlier stages of vocabulary development and it decreased with age.

That being said, there are certainly other studies that successfully demonstrated the effects of the quantity of ID speech on infants’ vocabulary size beyond 25 months, and one might wonder why the effect disappeared as early as 25 months in the current study. We speculate that there might be some variations in when the effect of the quantity of ID speech reaches its peak, depending on the particular design and methodology of studies. However, we would like to emphasize that, at least within our cohort of data that was specifically designed to observe the relative contributions of the three factors, infants’ own processing ability was a stronger predictor than the quantity of ID speech by 25 months. We suspect that infant’s processing ability would have remained stronger even if the quantity of ID speech continued to be significant by 25 months. Finally, one might wonder if it could be infants’ vocabulary size that determines the quantity of mothers’ speech. That is, infants with a larger vocabulary might prompt more speech from their mothers. However, previous studies have shown that this is not likely. The relative amount of mothers’ speech to children is quite stable and independent of listeners’ language levels (e.g., Smolak and Weinraub, 1983).

In keeping with previous studies showing correlations between infants’ ability to process speech and vocabulary development, the current study demonstrates that 19-month-olds who looked longer at the target in a word recognition test also had larger vocabularies at both 19 and 25 months. This raised the question of how infants acquire and develop their ability to process speech. One possibility is that the greater quantity of mothers’ speech helps infants develop more efficient speech processing skills by providing them with more exposure to words. For example, Hurtado et al. (2008) and Weisleder and Fernald (2013) showed that the amount of speech input infants were exposed to was positively correlated with the measures of infants’ speech processing efficiency. Furthermore, Fernald et al. (2006) showed that speed and accuracy in spoken word recognition at 25 months were correlated with measures of infants’ vocabulary size from 12 to 25 months. The efficient speech processing skills might enable infants to learn new words more quickly, which would in turn promote rapid vocabulary growth.

There were some limitations of this study that impact the ability to interpret results to the same level as other vocabulary development studies. First, the present study only had mothers to complete 15-min recording sessions, and only a portion of the sample was used in analysis. This is relatively brief sampling of maternal speech compared to other studies (e.g., Huttenlocher et al., 1991; Hart and Risley, 1995; Hoff and Naigles, 2002). However, compared to other studies that were specifically designed to explore the relationship between the quantity of mothers’ speech and infants’ language outcomes, our study examined potential influences of multiple factors on vocabulary development. Because our study involved more diverse data and greater number of measurements, we had to reduce the size of the recording data so that we can manage them within our limited resources (Note that other studies which examined multiple factors also tended to involve relatively shorter recording sessions around 15 min, e.g., Newman et al., 2016). Despite the small sample size of maternal speech, our results collectively suggest that there is a positive correlation between the quantity of mothers’ speech and infants’ vocabulary size earlier in development, a finding consistent to the previous reports. Nonetheless, the results for individual measures should be interpreted with caution. For example, no correlation was found between mothers’ MLU and infants’ vocabulary size in the current study. One intriguing possibility is that MLU, which is typically considered as a measure of syntactic complexity, has a lesser effect on infants’ lexical diversity. At the same time, Hoff and Naigles (2002) present compelling evidence for MLU by showing that mothers’ MLU accounted for the largest variance in children’s vocabularies. All in all, more studies are needed to determine the role of maternal MLU in infants’ vocabulary development, as the measure has been investigated relatively less compared to other measures of maternal quantity.

Second, it is worthwhile to note that the factors that were examined in the present study were able to account for 30% or less of the variance in infants’ vocabulary size at both ages. The results in the current study therefore raise many questions about other factors that may contribute to early individual differences in vocabulary development. Such factors might include children’s early vocabulary size (Marchman and Fernald, 2008), children’s production accuracy in word-initial position (Zamuner, 2009), children’s ability to repeat non-words (Stokes and Klee, 2009), neighborhood density and frequency characteristics of words that children use (Stokes, 2010), infants’ stable use of consonants (Majorano et al., 2014; McGillion et al., 2017), infants’ attention to the speaker’s mouth and gaze following (Tenenbaum et al., 2015), and the amount of adult-child conversational turns (Gilkerson et al., 2018). Furthermore, it would be interesting to know whether and how various other aspects of ID speech, such as mothers’ speech expressivity, communication style, and ability to establish joint attention, are related to their infants’ vocabulary development. Finally, as we had a somewhat homogeneous group of participants in this study, the socioeconomic status of the families was not investigated; this is also an obvious area for future research.

## Conclusion

The present study provides a more comprehensive view of the factors contributing to infants’ vocabulary size by investigating the relative contributions of the acoustic quality of mothers’ speech, the quantity of mothers’ speech, and infants’ ability to recognize words. These factors have been individually suggested to affect early vocabulary development in previous studies, but very few studies have considered their effects while controlling for possible effects of the other factors. Our results suggest that the quantity of mothers’ speech is a more powerful predictor of infants’ vocabulary size at the beginning of the word spurt, but infants’ own language abilities gain more importance as they progress in the vocabulary learning process. The comprehensive approach taken in the present study is an essential step toward enhancing our understanding of early vocabulary development. Investigating more extensive samples of parental input, families with diverse educational or social backgrounds, and other potential factors may enrich the current findings.

## Author Contributions

JS, KD, and JM developed the research questions and designed the study. JS conducted the experiments and coded the data. JS, KD, and JM analyzed the results and drafted the manuscript.

## Conflict of Interest Statement

The authors declare that the research was conducted in the absence of any commercial or financial relationships that could be construed as a potential conflict of interest.
